# Supplementary material for: Trends in mortality rates of cutaneous melanoma in East Asian populations
Source: PeerJ. 2016 Dec 22;4:e2809. doi: 10.7717/peerj.2809 (PMC5182992; doi:10.7717/peerj.2809)
Supplement: Supplemental Information 1 — Figures S1–8 showed the output results of joinpoint regression analyses for male and female in Japan (Fig. S1–2), Republic of Korea (Korea) (Fig. S3–4), China: Hong Kong (Hong Kong) (Fig. S5–6), and Singapore (Fig. S7–8). ASMR: Age-standardized mortality rates, per 100,000 using World standard population. The square points are ASMRs in specific year. Annual Percentage Changes (APC) are given as the legend. The mark of ∧ represents APC was significantly different from zero at a = 0.05. The 95% CIs were shown in Table 1. [file peerj-04-2809-s001.docx]

Supplemental Information 2

Displays of joinpoint regression plots for male and female in Japan, Korea, Hong Kong, and Singapore.

Figures S1-8 showed the output results of joinpoint regression analyses for male and female in Japan (Fig. S1-2), Republic of Korea (Korea) (Fig. S3-4), China: Hong Kong (Hong Kong) (Fig. S5-6), and Singapore (Fig. S7-8). ASMR: Age-standardized mortality rates, per 100 000 using World standard population. The square points are ASMRs in specific year. Annual Percentage Changes (APC) are given as the legend. The mark of ^ represents APC was significantly different from zero at *a*=0.05. The 95%CIs were shown in Table 1.


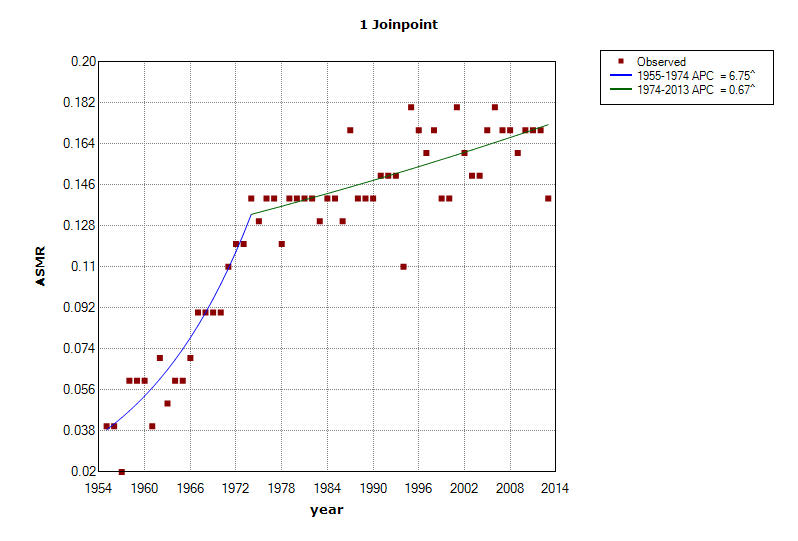


Figure S1: Joinpoint regression plot for female in Japan between 1955 and 2013.


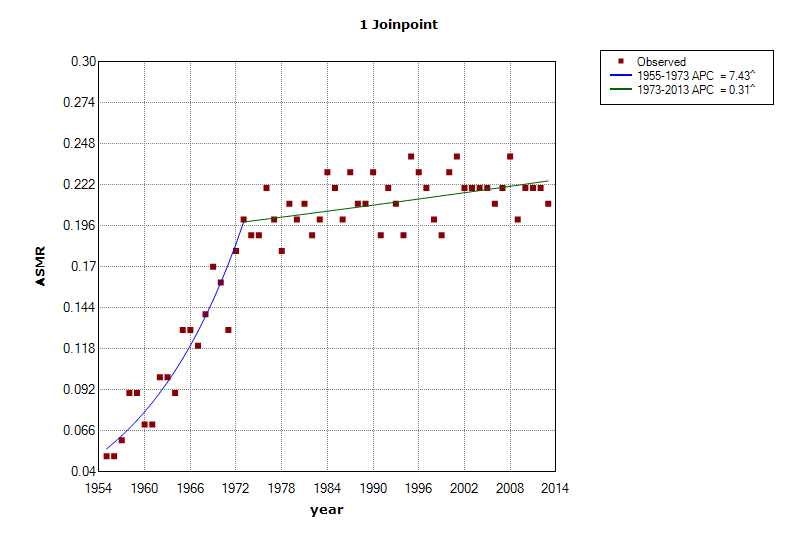


Figure S2: Joinpoint regression plot for male in Japan between 1955 and 2013.


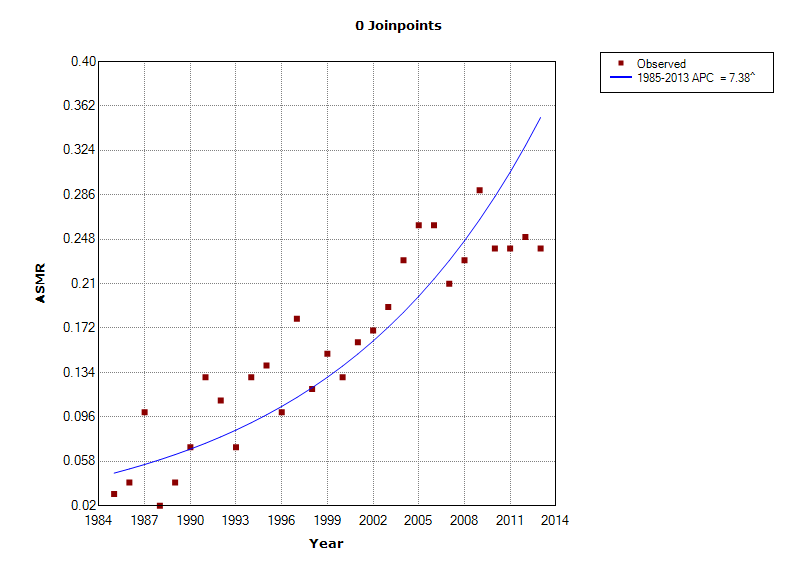


Figure S3: Joinpoint regression plot for female in Korea between 1985 and 2013.


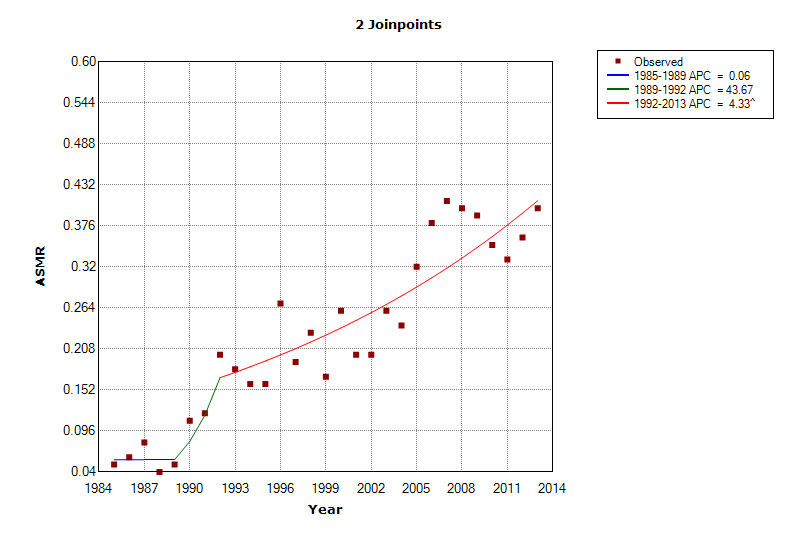


Figure S4: Joinpoint regression plot for male in Korea between 1985 and 2013.


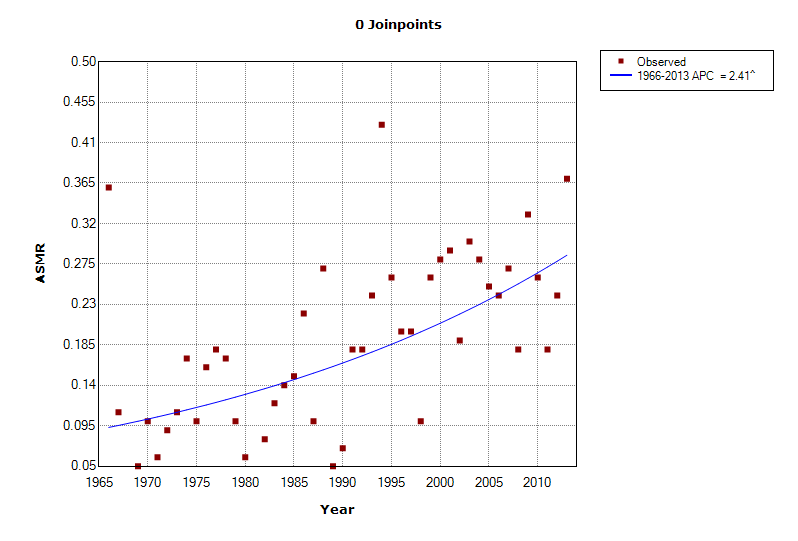


Figure S5: Joinpoint regression plot for female in Hong Kong between 1966 and 2013.


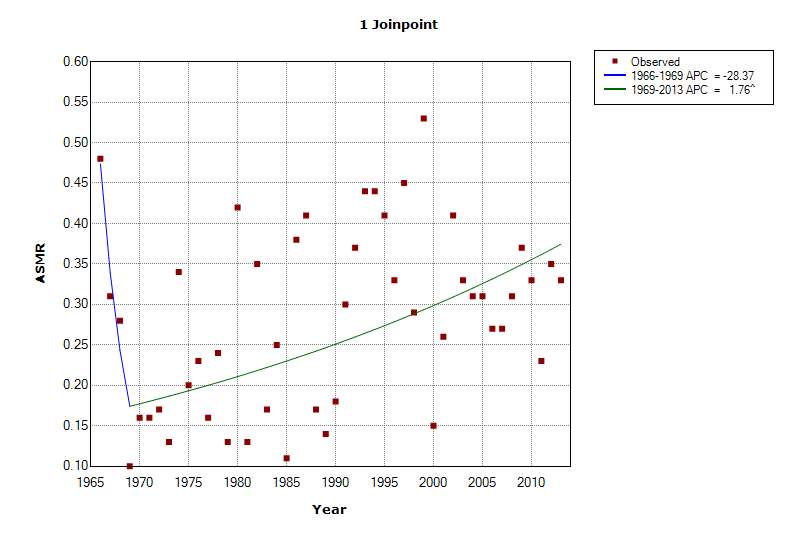


Figure S6: Joinpoint regression plot for male in Hong Kong between 1966 and 2013.


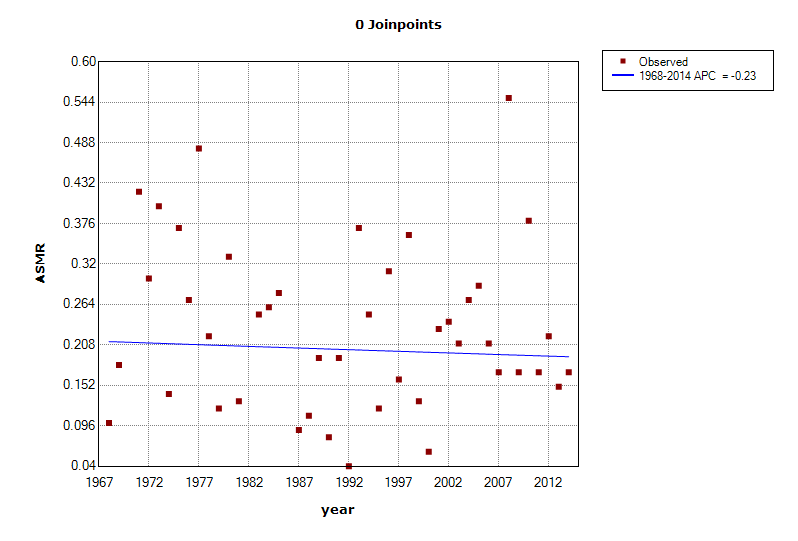


Figure S7: Joinpoint regression plot for female in Singapore between 1967 and 2014.


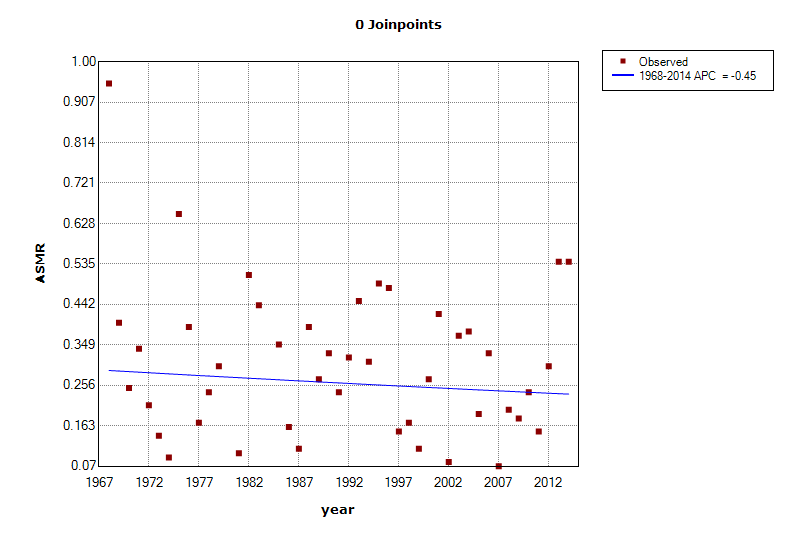


Figure S8: Joinpoint regression plot for male in Singapore between 1967 and 2014.
