# Supplementary material for: Trends in mortality rates of cutaneous melanoma in East Asian populations
Source: PeerJ. 2016 Dec 22;4:e2809. doi: 10.7717/peerj.2809 (PMC5182992; doi:10.7717/peerj.2809)
Supplement: Supplemental Information 2 — Figure S1: Smooth curve of year on the age-standardized mortality rates (ASMRs) in the four East Asia regions between 1955 and 2014. The x-axis shows the value of years recorded, the y-axis shows the ranges of partial residual for the term of year. The solid line is the results by smoothing the partial residual for the term of year estimated; the shade region represents the 95% confidence intervals (95% CI) of the smooth curve. The value of the estimated degrees of freedom (edf) is showed in the parenthesis. The edf, ranged zero and infinity, was used to express the extents of non-linear of smooth curve. The higher the edf, the more non-linear trends in ASMRs over the years were obtained. The part of the s(year) in the (1) is a corresponding smooth function by iteratively smoothing partial residual using backfitting algorithm. The partial residual for the ith (here, the ith year) term was calculated by subtracting all estimates of terms from the response variables, except for that of the ith term (Wood, 2006). Therefore, this figure is substantially a partial residual plot, which can show the relationship between a given independent variable (here, year,) and the response variable (here, age-standardized mortality rates) assuming there are other independent variables. Overall, the ASMRs of East Asia population had a significant non-linear growth over the past six decades (edf =3.9, P < 0.001 for the year term in GAM). [file peerj-04-2809-s002.docx]

**Trends in age-standardized mortality rates in East Asians between 1955 and 2014.**


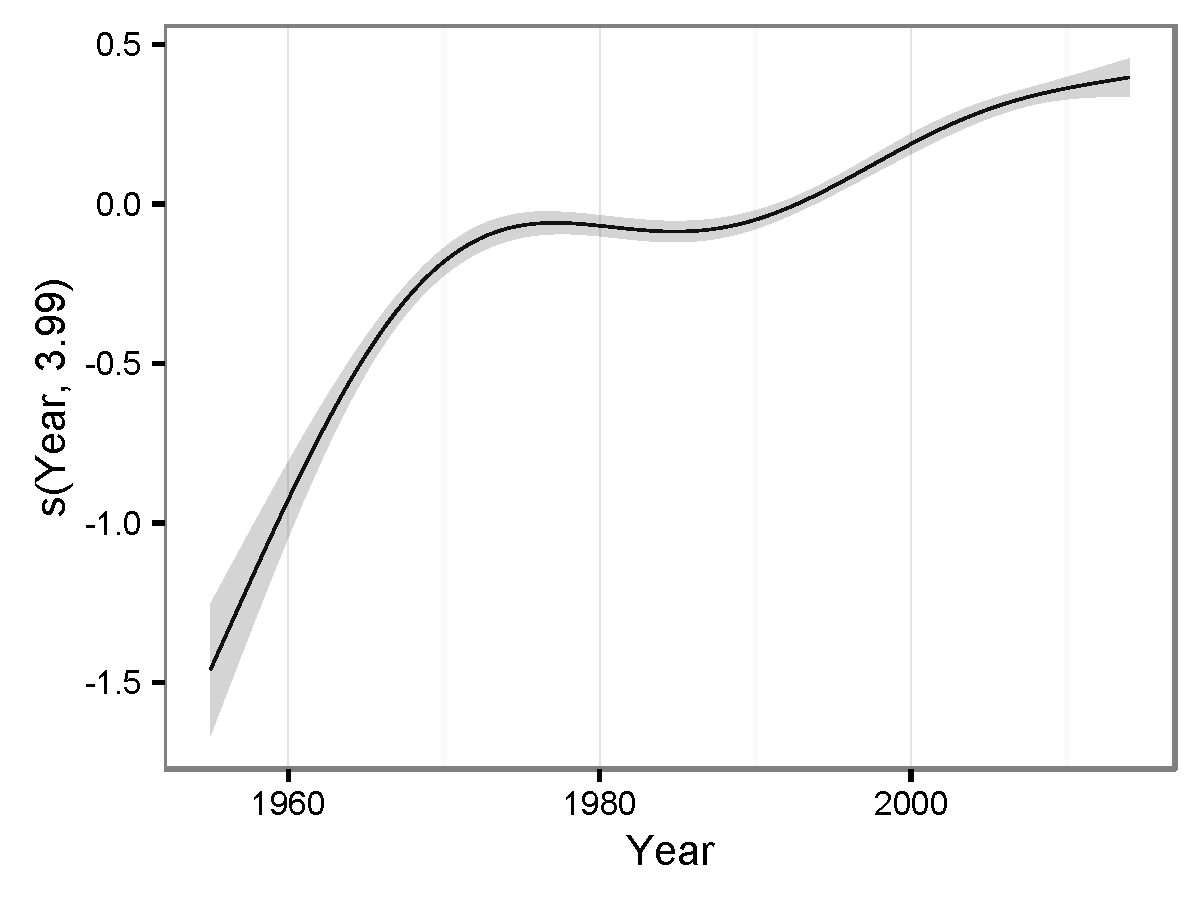


Figure S1: Smooth curve of year on the age-standardized mortality rates (ASMRs) in the four East Asia regions between 1955 and 2014. The *x*-axis shows the value of years recorded, the y-axis shows the ranges of partial residual for the term of year. The solid line is the results by smoothing the partial residual for the term of year estimated; the shade region represents the 95% confidence intervals (95%CI) of the smooth curve. The value of the estimated degrees of freedom (edf) is showed in the parenthesis. The edf, ranged zero and infinity, was used to express the extents of non-linear of smooth curve. The higher the edf, the more non-linear trends in ASMRs over the years were obtained. The part of the s(year) in the Equation (1) is a corresponding smooth function by iteratively smoothing partial residual using backfitting algorithm. The partial residual for the *i*th (here, the *i*th year) term was calculated by subtracting all estimates of terms from the response variables, except for that of the *i*th term (Wood, 2006). Therefore, this figure is substantially a partial residual plot, which can show the relationship between a given independent variable (here, year,) and the response variable (here, age-standardized mortality rates) assuming there are other independent variables. Overall, the ASMRs of East Asia population had a significant non-linear growth over the past six decades (edf=3.9, *P* < 0.001 for the year term in GAM).
